# Women's health, hormonal balance, and personal autonomy

**DOI:** 10.3389/fmed.2023.1167504

**Published:** 2023-06-30

**Authors:** Ignacio Segarra, Micaela Menárguez, María Victoria Roqué

**Affiliations:** ^1^Department of Pharmacy, Faculty of Pharmacy and Nutrition, Catholic University of Murcia (UCAM), Murcia, Spain; ^2^“Pharmacokinetics, Patient Care and Translational Bioethics” Research Group, Faculty of Pharmacy and Nutrition, Catholic University of Murcia (UCAM), Murcia, Spain; ^3^Bioethics Chair, Faculty of Medicine, Catholic University of Murcia (UCAM), Murcia, Spain

**Keywords:** contraception, health care delivery, medical humanities, women's health, autonomy, adverse effects

## Abstract

Hormone-based contraception disrupts hormonal balance, creating artificial states of anovulation and threatening women's health. We reviewed its main adverse effects and mechanisms on accelerated ovarian aging, mental health (emotional disruptions, depression, and suicide), sexuality (reduced libido), cardiovascular (brain stroke, myocardial infarction, hypertension, and thrombosis), and oncological (breast, cervical, and endometrial cancers). Other “collateral damage” includes negative effects on communication, scientific mistrust, poor physician–patient relationships, increased patient burden, economic drain on the healthcare system, and environmental pollution. Hormone-sensitive tumors present a dilemma owing to their potential dual effects: preventing some cancers vs. higher risk for others remains controversial, with denial or dismissal as non-relevant adverse effects, information avoidance, and modification of scientific criteria. This lack of clinical assessment poses challenges to women's health and their right to autonomy. Overcoming these challenges requires an anthropological integration of sexuality, as the focus on genital bodily union alone fails to encompass the intimate relational expression of individuals, complete sexual satisfaction, and the intertwined feelings of trust, safety, tenderness, and endorsement of women's femininity.

## 1. Introduction

If an event in nature abides by perfect synchronization to ensure a successful outcome, it is reproduction. All female physiological and biochemical processes related to fertility and the possibility of pregnancy are highly regulated, maintaining a precise hormonal balance throughout the duration of a menstrual cycle (1–3). The synchronized hormonal secretion along the menstrual cycle triggers the physiological conditions of the endometrium to receive the fertilized ovum and provide “food and shelter” to allow it to reach maturity (4–6).

When pregnancy, lactation, or menopause are not the causes of persistent irregularities in the menstrual cycle, it is likely that they are associated with stress and lifestyle, endocrine disorders, gynecological, nutritional, genetic, and even iatrogenic factors (7). This situation may disrupt the precise hormonal balance, giving rise to pathological situations that may deserve medical attention, including polycystic ovary syndrome, diabetes, Cushing's syndrome, and hypothyroidism (3). Therefore, a regular menstrual cycle is an indicator of health in women (6). Conversely, some factors facilitate regulation and normal ovulation (e.g., healthy behavior and lifestyle, calm in the face of stress, restoration of normal conditions of pathological processes, and so on), including the evaluation of specific personal needs (7).

Thus, any interference with the hormonal cycle would prevent either ovulation or implantation and constitute the pharmacological basis for all hormone-based contraceptive (HBC) methods. Therefore, understanding hormonal regulation and the evolution of the ovaries is essential to assessing the impact of HBC on women's health, including oocyte aging and the ovary reserve. When the development of physiology follows its natural course, organs or tissues evolve over time, affecting their functionality. If any tissue or organ undergoes intervention, it can be affected either positively (to restore lost functionality due to a pathology) or negatively (as trauma or infection may reduce the functionality of that organ).

The ovaries also undergo some stages that determine their functionality (8). During childhood, the follicles develop progressively, and it is not until puberty that an increase in gonadotropic hormones occurs, giving rise to a pre-ovulatory state and the first ovulation at around 12–13 years of age (8, 9). Until then, anovulation is a normal manifestation of a girl's health. From that moment onward, cyclical ovarian activity begins, which may present some irregularities until the woman is approximately of 18 years of age. During adolescence, menstrual cycle irregularities are considered healthy and normal, while the hypothalamic-pituitary axis activity gradually increases until it becomes regular, which is typical of a woman's fertile age. This cyclical activity remains regular in healthy women until ovarian functionality decreases, as does the hypothalamic-pituitary axis activity, which gradually enters a pre-menopausal period that may last several years. During this period, there is an increase in estrogen levels, promoting the growth of endometrial tissue associated with increased blood flow, irregular bleeding, and the loss of fertility, giving way to menopause.

The aging of the ovaries in women follows normal organ evolution, reducing the quantity and quality of the oocytes (10–12). A unique physiological feature of ongoing change is the “ovarian reserve,” or the ovarian capacity to generate ovules that can be fertilized (13–15). This capacity can be assessed by biomarkers that indicate the status quo of the ovaries: antimulerine hormone (AMH), antral follicle count (AFC), and ovary volume. The follicles secrete AMH, and its serum value reflects how many valid eggs remain in the woman's ovaries (16) regardless of the woman's fecundability (17); the AFC and the volume of the ovaries are determined by ultrasound. These biomarkers assess the ovarian status to evaluate fertility problems in women who use or have used oral contraceptives (12, 18–20).

All this information places a responsibility on healthcare providers, including the pharmaceutical industry (21), to better understand the impact of HBC, provide adequate information to women users, and empower them to lead a healthy and satisfactory sexuality of their choice.

### 1.1. Historical context: medicine, culture, and society

The first HBC introduced in the late 1950s combined high doses of estrogen and progestogen but was eventually modified (22). Current HBCs have lower doses and introduce temporality through biphasic and triphasic formulations (23, 24). This reduction strategy aimed to minimize adverse effects on lipid metabolism (obesity, accumulation of trunk fat characteristic of men, and high levels of cholesterol and triglycerides), carbohydrate metabolism, including insulin resistance and diabetes (25), homeostatic parameters associated with cardiovascular risks (26), and to produce effective control over the ovulatory cycle. A later technological step would introduce chemically structural analogs that, instead of preventing ovulation and fertilization, would prevent nesting and implantation of the fertilized ovum, causing an induced abortion, e.g., emergency postcoital contraception (27, 28) or self-induced abortion (29, 30). However, their assessment is beyond the scope of this review.

Thus, the physiological objective of current HBC is to create an artificial situation of anovulation by altering the hormonal balance and suppressing the ovulatory cycle to prevent fertilization and minimize the risk of adverse effects associated with the administration of estrogen derivatives such as ethinyl estradiol (EE). The actual rate of HBC use discontinuation reaches 59%, of which 61% is attributed to adverse effects on women's health (22).

Therefore, a review of the potential effects and their impact on women's health may provide a better understanding of women's needs.

## 2. Adverse effects

The adverse effects of HBC have been one of the most controversial issues in the last 40–50 years of health care. A recent review summarized their main adverse effects and other features, such as their communication (31). Besides the generally recognized adverse effects (breast and other cancers, emotional and psychiatric disorders, cardiovascular risks, and so on), other adverse effects that are not usually acknowledged and risk being underreported include increased risk of HIV transmission, immunology disorders (Crohn's disease, ulcerative colitis, lupus erythematosus), “suicide, multiple sclerosis, interstitial cystitis, female sexual dysfunction, bone fractures, and increased fat mass.” The authors also identified adverse effects for which medical information provided to the user is usually biased: cardiovascular risks (heart attack, stroke, and thrombosis) (31).

### 2.1. Ovarian aging

Ovarian functionality can be objectively assessed through three biomarkers: ovary volume, AMH, and AFC. The AMH serum concentration is a reliable predictor of ovarian aging approaching menopause (32, 33) to evaluate a woman's fertility (17, 18, 20). A study analyzing the ovarian reserve (16) showed that combined HBC, as compared to progestin-only HBC, leads to a statistically significant decrease in AMH levels (−31.1 and −35.6%); AFC (−31.3 and −29.7%); and ovarian volume (−57.2 and −10.5%), respectively. Intrauterine systems and vaginal rings had less pronounced effects: −17.1 and −12.2% for AMH, respectively; −5.9 and −22.7% for AFC, respectively; their effect on the ovarian volume was highly dependent on the method: the intrauterine system caused a −5.1% decrease, while the vaginal ring resulted in a −55.8% decrease (16). These results validate other studies where AMH, AFC values, and the volume of both ovaries were significantly decreased by 19%, 18%, and 50%, respectively, in HBC women users (18, 20). A relationship between HBC use duration (as well as tubal ligation) and lower AMH serum levels was found to be statistically significant (*p* = 0.036) and was independent of the age of first use of contraception (34), although this may not necessarily lead to early menopause (35).

Ovarian aging may also be studied through the evolution of cervical mucus. Various types of cervical mucus are secreted by specific S, L, and G glands in the cervix (36, 37). Their percentage varies with the phases of the menstrual cycle to facilitate or prevent the sperm from reaching the ovum (38, 39), similar to pregnancy or menopause (40), and deviations from their cyclical variation may show underlying problems of ovulation (38). These variations are also associated with age (41–43): the number of S-type glands decreases at an estimated 2% rate since adolescence and is replaced by type L starting from the base of the cervix (42). Pregnancy seems to exert protection against further replacement of mucus types S and L by G in the cervix due to a lower decrease rate of 1.2% or even rejuvenating the cervix, an equivalent of 2–3 years (42). However, HBC causes biochemical changes in the mucus composition (44–46) and favors its replacement by G type at a 4% rate (faster than the natural aging process regardless of pregnancies), causing a lower mucus score due to cervical atrophy lacking functionality (41, 47–49).

The changes in serum biomarkers and cervical mucus could manifest as accelerated ovarian aging. Women using HBC for 10 years may find themselves hindering the possibility of later pregnancies due to loss of functionality (42, 50) and difficulties in reestablishing fertility after HBC discontinuation (18, 35, 51).

### 2.2. Effects on mental health

The psychological effects associated with HBC are one of the main causes of dissatisfaction and discontinuation (22, 52). They include a variety of neuro-bio-psychological scenarios of different severity: behavioral changes, emotional and affective changes, anxiety, depression, suicide attempts, and suicide.

#### 2.2.1. Psychological effects: affective and emotional disruptions

The first studies that observed these changes dating to the 1980s, described diagnosis rates between 20 and 50% (53). Novel approaches to HBC combined with 35 μg of EE led 47% of women to discontinue after 1 year, citing emotional and affective adverse effects in 33% of women; only 27% continued after 6 months due to mood effects (53). Comparison studies concluded that women using the vaginal ring vs. orally administered HBC had fewer negative emotional changes (54), less irritability, depression, and emotional variability (55), similar to those found in women using HBC transdermal patches (56). However, caution is needed. Some articles compare different HBC formulations without control groups, making interpretation complex (53).

Evaluation of psychotropic drug use validated the impact of HBC on mood. A study carried out in Finland (57) compared HBC women users to never users (*n* = 294,356 women per group) and analyzed their psychotropic drugs. The results showed HBC female users had a moderately higher relative risk (RR) ranging from 1.1 to 1.3 for all psychotropic drug classes (*p* < 0.0001): antipsychotics, anxiolytics, hypnotics/sedatives, antidepressants, and a combination of psycholeptics with psychoanaleptics, except for psychostimulant drugs, and a higher incidence of psychotropic drug use was found in adolescent women (57).

Women with a clinical history of adverse emotional effects from HBC, who were re-exposed to EE combined with levonorgestrel, exhibited emotional deterioration compared to the control group (who received a placebo), where no emotional change was observed. The group that received HBC also showed a lower induced emotional response in the brain areas associated with emotion recognition and regulation (58–61). Further studies in young women before and after starting HBC showed a lower volume of the gray matter in the amygdala, the parahippocampus, and the connectivity between the amygdala and the prefrontal cortex (62), highlighting the need to research HBC-induced brain changes.

Mechanistically, the synthetic hormonal analogs cross the blood–brain barrier, reach the receptors, develop their effects in the central nervous system (63–65), and induce psychological outcomes through biochemical-neurological mechanisms (66). Sex hormones exert their effects on the human brain through estrogen receptors in areas involved in the regulation of emotions (the amygdala and the hippocampus), affecting the synthesis of neurotransmitters associated with emotions, serotonin, and γ-aminobutyric acid (GABA) (67–70). Moreover, the duration of HBC use correlates with changes in the volume of the gray matter in the hippocampus, the cerebral basal nuclei (71), the amygdala, and the *nucleus accumbens*. This finding is particularly relevant: the effects seem more severe if HBC is used during adolescence (72, 73); they are not immediately reversible after discontinuation, and a link with depression, the prescription of psychotropic drugs, and antidepressants seem to exist (74). In addition, emotional disorders exhibit heightened severity in women who had pre-existing emotional conditions prior to using HBC, as well as in adolescent women, due to their vulnerability (68).

#### 2.2.2. Effects on sexual life

The effect of hormonal changes caused by the HBC on women's sexual lives and man–woman relationships throughout the menstrual cycle was evident.

“…increases in estradiol negatively impacted women's and men's romantic relationships. Specifically, as estradiol increased, women evaluated their partners less positively, and they were less physically attracted to their partners. […] Increases in progesterone (which peaks after ovulation) were associated with more positive perceived relationship evaluations and personal wellbeing in women” (75).

The decreased reactivity against negative stimuli and the constant presence of estrogen derivatives (mainly EE) could cause emotional destabilization at the psychological and behavioral levels. In addition, combined HBC increased SHBG serum levels and decreased the androgen hormone testosterone regardless of the progestin and estrogen doses used (76), with high SHBG serum levels after 120 days post-discontinuation (77). These changes cause a loss of sexual drive and libido, affecting a woman's sexual life (76–79). Other studies have also shown negative effects on women's sexuality, and controversy included: “attributing a sexual dysfunction to the use of a contraceptive is to be avoided at all costs,” although acknowledging that “contraceptives could also cause a sexological disorder” (80) or sexual dysfunctions in women (78, 81–83).

#### 2.2.3. Depression

Recent studies have used sex hormones to identify the underlying mechanisms leading to depression and ways to prevent it, including modification of serotonin transport (84, 85). Thus, it is not surprising that HBC may induce depression (52).

In a study including 815,662 women (12–30 years old), 3.1% of the participants were dispensed psychotropic drugs (52), which was 3.7% and 2.5% for HBC users and non-users, respectively, with an RR of 1.34 (95% CI: 1.30–1.37). The risk of psychotropic drug use was greater in women aged 12–20 years, with a 4–3.5% range in users vs. < 2% in non-users, and it was >4% (users) vs. 0.9% (non-users) in 12–14-year-old women with RR = 3.46 (95% CI: 3.04–3.94) or 4.47 (95% CI: 2.08–8.78) for progesterone-only formulations (intravaginal ring and transdermal patch) (52). These results agree with other studies that found an association between HBC use and the diagnosis of depression in women (particularly in women aged 12–19 years old) with a RR of 1.12 (95% CI: 1.05–1.19) vs. non-users (86, 87). Both studies have discovered that premature HBC use involves serious risks for women's mental health, including the impact on the adolescent's affectivity and future sexual relationships (88). This adverse effect seems associated with enzyme monoamine oxidase deregulation due to high estrogen and progesterone concentrations (89, 90).

#### 2.2.4. Suicide risk

The most serious HBC adverse effect that has attracted the most attention is suicide and attempted suicide (91–93). A study carried out in Denmark (1996–2013) with 475,802 women identified as HBC users aged 15–33 years had an RR of 1.97 (95% CI: 1.85–2.1) for a first suicide attempt and 3.08 (95% CI: 1.34–7.08) for suicide (91). Young women (15–19 years) were the most susceptible, with an RR of 2.06 (95% CI: 1.92–2.21), highly significant (*p* < 0.0001); this RR was 2.5 after 1 month of HBC use, remained above 2 during the first year, and was 30% higher after 7 years. These findings corroborate another study with women aged 15–22 years: the suicidal behavior RR ranged from 1.56–2.13 1 month after beginning HBC use and 1.19–1.48 after one year (92). Further evidence on the higher suicide risk associated with HBC shows an RR of 1.36 (95% CI: 1.06–1.75) (94), 1.23 (95% CI: 1.1–1.37) in a Korean study (95), and others (96).

Discontinuation of HBC use did not eliminate the RR of first attempt suicide (RR = 3.4, CI: 3.11–3.71) or suicide (RR = 4.82, CI: 1.93–12.1) in women who had previously used HBC, with combined estrogen-based vs. progestin-only formulations having a similar impact (91). Although all HBC types increase suicide or suicide attempt risks, norelgestromin patches and implants increase them by 3.9 (95% CI: 2.48–6.14) and 5.85 (95% CI: 4.80–7.13), respectively, and medroxyprogesterone acetate implants by 10.2 (95% CI: 7.87–13.2).

Other studies seem to contradict these risks. Usually, these studies lack coherent longitudinal analysis or experimental design. A suicide RR reduction has been suggested, but the study was designed to evaluate depression, lacking appropriate population selection (97); similarly, HBC users showed no higher RR, but it was limited to 12 years and assessed general mortality rather than suicide (98). In other cases, the HBC effect cannot be determined because of confounding factors and population bias: study and control groups both use HBC, although different classes and mortality associated with other pathologies (ovarian, cervical cancer, and cardiovascular diseases) cannot be isolated from HBC use (99). This variability between studies seems to be a consequence of the “low or moderate methodological quality of the study” (100), including low statistical power to identify an effect (101, 102), overlap and redundancy of factors and variables (103), poor demographic data (97), high group heterogeneity (102), or drawing conclusions beyond the study design (98, 99, 101, 104, 105).

### 2.3. Increased cardiovascular risk

Cardiovascular risk is one of the best-studied adverse effects of HBC due to its ability to interact with estrogen and progesterone receptors present in the tissue layers of the blood vessels (106). Several studies have recognized the association between HBC and increased risk of venous or arterial thrombosis (106–113), brain stroke (114–117), myocardial infarction (118), and hypertension (119–121).

Studies carried out by the WHO showed higher thromboembolism RR associated with duration, type, and dose: one-year users had RR = 5.63, which remained >3 up to 8 years (108); the overall RR was 4.1 (95% CI: 3.2–5.2), 9.1 (CI: 4.9–17.0) for desogestrel, and 9.1 (CI: 4.9–16.7) with gestodene (110); it was dose-dependent, with twice the risk at 50 μg (106). In third-generation HBC, the thrombosis RR was 3.6 (95% CI: 2.9–4.6) for levonorgestrel, 5.6 (95% CI: 3.7–8.4) for gestodene, 6.3 (95% CI: 2.9–13.7) for drospirenone, 6.8 (95% CI: 4.7–10) for cyproterone acetate, and 7.3 (95% CI: 5.3–10) for desogestrel (106), suggesting that the third-generation HBC did not eliminate the risks. Furthermore, the RR increased synergistically (12–24 times) in women with additional risk factors, e.g., obesity (111–113). The vascular damage may be related to changes caused by EE in the homeostatic chain of coagulation (109): higher generation of thrombin and coagulation factors (fibrinogen, VII, VIII, IX, XII, XIII) and reduction of coagulation inhibitors (protein C, antithrombin). These actions could lead to cardiovascular complications, including thrombus formation. Third-generation HBC may have aggravated the problem due to increased resistance to protein C, confirming the findings of the WHO (106).

A higher incidence of cerebral stroke among HBC users compared to non-users has also been shown. For doses of EE >50 μg, the RR was found to be 5.3 (95% CI: 2.6–11), while for doses below 50 μg, the RR was 1.53 (95% CI: 0.71–3.31) (114). Additionally, HBC containing low doses of norgestrel or levonorgestrel (117) showed an increased RR for hemorrhagic stroke at 3.23 (95% CI: 1.24–8.41), and the RR for aneurysmal bleeding increased to 4.46 (95% CI: 1.58–12.53). Similar results were found in a study addressing the role of migraine, and the risk of cerebral stroke RR was 2.52 (115) in patients with and without aura, increased to 6.25 and 6.35, respectively (116), and was dose-related (122). Furthermore, the data suggest that the stroke RR in HBC users is affected by the presence and degree of other pathologies (115, 123).

Myocardial infarction and hypertension may also be mentioned. The RR of myocardial infarction increased to 2.48 (95% CI: 1.91–3.22) in HBC users vs. never users and remains moderate after discontinuation at 1.15 (95% CI: 0.98–1.35) without its complete disappearance (118). A 43% higher prevalence was observed in postmenopausal women who used HBC for over 30 months (124).

## 3. Oncology-related adverse effects

The incidence of various types of cancer has spurred research into their generation and progression mechanisms, revealing the role of hormones in their development (125). Specifically, types of cancer that are estrogen dependent are characterized by tumor cells that possess estrogen receptors and benefit from the hormone estrogen to progress.

Thus, antiestrogen endocrine therapy may block their growth, while progesterone-dependent types of cancer rely on the sensitivity of cancer cells to progesterone for growth, which can be blocked through endocrine hormonal treatment. On the other hand, non-dependent types of cancer lack hormone receptors, and their growth remains unaffected by hormones.

### 3.1. A therapeutic-based controversy

The identification of hormone-sensitive tumors has given rise to one of the most debated scientific issues over the years: whether HBC may increase the risk of developing some types of cancer in women (126–129). The rationale behind this is that most HBC formulations are analogs or identical to estrogens or other hormones that can affect tumor progression. This question has sparked much controversy in the interpretation of data, with assertions of cancer protection on one hand and denial of scientific evidence on the other, likely influenced by the desire to alleviate concerns surrounding HBC usage (82, 130–134), biased data analysis, and inadequate selection of study populations (135, 136).

Their potential dual effects present a therapeutic dilemma (135): higher RR of specific kinds of cancer (e.g., breast cancer) vs. protective effect against other types of cancer (e.g., endometrial cancer). The authors proposed their use in women who have been or are undergoing cancer treatment but also stated a lack of evidence to draw valid conclusions. An analysis of their methodology shows a lack of population selection since the study population (women with cancer) would not represent the general population. Given their study limitations, the authors appropriately warned against using HBC due to possible effects on hormone-sensitive tumors.

Another study concluded that HBC users would not have a greater long-term cancer risk but rather a reduced risk and protection against some cancers (136). Similarly, a lack of population selection leads to that interpretation: first, women who had cancer before the start of the study (even if they used HBC) were excluded; second, the stratification of HBC users vs. non-users was not well established; third, there was a notable lack of study follow-up (53% of participants stopped providing the information); fourth, only the first cancer cases were counted and subsequently censored. This meant that the sample size of HBC users who had cancer progressively decreased and, compared to non-HBC users, yielded an apparent protective effect. Nevertheless, the authors reported a RR 5 years after HBC discontinuation of 2.33 (95% CI: 0.43–12.6) for pancreatic cancer, 1.48 (95% CI: 1.10–1.97) for breast cancer, 2.32 (95% CI: 1.24–4.34) for invasive cervix cancer, 2.20 (95% CI: 0.49–9.99) for central nervous system cancer, and 1.45 (95% CI: 0.14–14.8) for thyroid cancer. Regardless of the methodological pitfalls, both studies put forward evidence of a higher cancer incidence in HBC users.

### 3.2. Breast cancer risk

Initial, old studies calculated a worrying increase in breast cancer RR up to 40% for women aged 20–40 (137), 88% (138), and 42% (139), and 50% RR if HBC were used within 5 years of menarche (140, 141). Overall, these studies did not address mechanisms, lacked a significantly large study population (unlike later longitudinal studies), and presented methodological errors, e.g., mixing populations of users and non-users in control groups (140). More recent studies include a mechanistic scope and have confirmed the influence of estrogens in the regulation dynamics of the transcription process (142, 143) as well as genes involved in tumor cell proliferation, especially levonorgestrel, desogestrel, and gestodene (144). This recent evidence puts forward the need for an in-depth analysis of the effects of HBC on breast cancer (145).

In a study involving 1.8 million women over nearly 11 years, the breast cancer incidence RR was 1.20 (95% CI: 1.14–1.26) for HBC women users (146). The RR was found to be 1.09 (95% CI: 0.96–1.23) for users of HBC for < 1 year and 1.46 (CI 95%: 1.32–1.61) for users for more than 10 years. The RR remained 10 years after discontinuation of HBC, and it was >2 in women who had used it for more than 10 years. However, the specific magnitude of the relative risk varied depending on the type of HBC used. Other studies have provided further confirmation, indicating a RR of 1.2 (95% CI: 1.14–1.26) for breast cancer among HBC users (147). Specifically, levonorgestrel-releasing intrauterine systems were associated with a RR of 1.16 (95% CI: 1.06–1.28). Notably, the RR differed among age groups, with a value of 1.12 (95% CI: 1.02–1.22) for women under 50 years of age and 1.52 (95% CI: 1.34–1.72) for women above 50 years of age, suggesting an underlying risk that may increase over time (148).

A causal relationship has been proposed between HBC containing EE and the development of breast cancer RR (142, 143). However, it appears that these scientific findings are often overshadowed by the perceived benefits of HBC and are only considered in particular cases. “These data should be brought to light in view of the great benefit of hormonal contraception in the female reproductive context” (149) while also acknowledging the potential adverse effects that may have been overlooked or disregarded (150–152).

### 3.3. Risk of cervical cancer

HBC disrupts the balance of estrogen, inducing artificial cell changes. There was a significant correlation (*p* < 0.01) between the duration of HBC use and the incidence of cervical cancer, with an overall RR of 4.2 (95% CI: 1.01–5.69) when its use exceeded 5 years, which reached 7.1 (95% CI: 1.74–28.9) for oral formulations (153); these findings are consistent with those from previous studies: RR was found to be 1.1 (95% CI: 1.1–1.2), 1.6 (95% CI: 1.4–1.7), and 2.2 (95% CI: 1.9–2.4) for < 5 years, 5–10, and more than 10 years of HBC use, respectively (154). Similar outcomes were observed in women aged 15–49 years in 1995–2014, with an overall RR of 1.19 (95% CI: 1.10–1.29), which increased to 1.40 (95% CI: 1.28–1.53) with duration and combined type (155). In addition, the authors identified other relevant items: first, the RR in long-term HBC users requires over 10 years to disappear post-discontinuation and remains 1.29 (95% CI: 0.65–2.56); second, the new EE-norethisterone combined formulations present the same risk as previous formulations, a RR of 2.68 (95% CI: 1.68–4.28), which was expected since both formulations have the same active components; and third, women need to be informed correctly about the risks to make an informed decision or choose other alternatives (155).

### 3.4. Risk of endometrial carcinoma

Endometrial carcinoma cells exhibit estrogen receptors, and their growth is stimulated by estrogens (156). Mechanistically, preclinical studies have shown that the metabolite of estradiol, 4-hydroxy-estradiol, induces DNA damage in endometrial cells (157, 158). Moreover, other metabolites (e.g., 17α-ethinylestradiol) increased the incidence of uterine adenocarcinoma (159, 160). Additionally, changes in endometrial morphology, histology, and functionality have also been associated with levonorgestrel-releasing intrauterine devices (161–163). An increase in endometrial cancer risk may be related to the hormonal balance of estrogen and progesterone concentrations modulating the endometrial mitotic activity rate changes during the menstrual cycle (164). Interferences could cause deregulated mitotic activity and a higher risk of endometrial cancer, which even led to the removal of some formulations from the market (165).

Endometrial carcinogenic damage associated with combined HBC has been reported in clinical studies (165–167) and with lesser evidence in case series (168–174). Endometrial cancer RR of 1.36 (CI 95%:0.39–4.70) was calculated for women with a BMI < 22.1 kg/m^2^ vs. 0.31 (95% CI: 0.11–0.92) in women with a BMI >22.1 kg/m^2^ (166). Obesity largely modifies the volume of distribution of lipophilic drugs due to their high affinity for the adipose tissue, leading to the removal of the drug from the bloodstream and its accumulation in the adipose tissue. This would provide a pharmacokinetic-based explanation of the 30% lower plasma concentration observed in obese women (175) and the greater failure rate of HBC in obese women (176, 177). Thus, women with a BMI above 22.1 kg/m^2^ who have low HBC bloodstream concentrations would be at lesser risk of endometrial cancer and significant adverse effects. To avoid a high rate of contraceptive failure, women with a high BMI were systematically excluded from clinical studies (178).

Although the assessment of HBC's protective effect exceeds the goals of this review, it is important to point out that several studies (with diverse quality) have indicated protection against endometrial cancer (136, 165, 179, 180), not without disdain for potential adverse effects (181–183). An endometrial cancer risk evaluation based on formulation type concluded a protective effect with an RR range of 0.94–0.37 (167). However, this study presents a bias in the population selection: the control group includes never HBC users together with women who had used other methods such as a diaphragm or intrauterine device (18.1%), male contraception (32.7%), tubal ligation (9.8%), or others (23.6%). Thus, no conclusion may be drawn since some of those other methods may affect hormonal balance.

However, even if these benefits were fully proven in *ad hoc* clinical trials, their impact on other women's health aspects cannot be overlooked, lest other adverse effects appear upon the preventive treatment of endometrial cancer (123). Conversely, there is clear evidence of a 40% reduction in endometrial cancer in parous vs. nulliparous women, probably due to progesterone's protective effect on the endometrium (184, 185).

## 4. Collateral adverse effects

Other general aspects related to women's health are also negatively affected, including a lack of information about adverse effects provided to current and new HBC users at prescription or dispensing levels; degradation of the patient-healthcare professional relationship; scientific relativism with two-tier validity criteria and language change; the impact of contraception on individuals and society at large; and the degradation of the environment.

### 4.1. Patient information delivery

Accurate and timely information on HBC is necessary to guarantee an informed decision and ensure good clinical outcomes. Studies examining the provision of information to women regarding cardiovascular risks associated with HBC have indicated inadequacy. The emphasis is often placed on the efficacy and utilization of HBC, with minimal attention given to the possible adverse effects and their reversibility (155). The seriousness of some adverse effects may influence their decision to use HBC (22, 54, 83, 186), and it could also affect a woman's physiology capacity to restore her normal physiology (51, 124) and fertility after experiencing years of hormonal imbalance (18, 19, 134, 187). Furthermore, women with pathologies could undergo more severe adverse effects, potentiation of the pathology (111–113, 115, 118, 124, 177), or undesired sequelae (155, 188–191). Thus, it is worth stressing that women with cardiovascular pathologies, such as diabetes, depression, and polycystic ovary syndrome, are particularly vulnerable and require greater attention to mitigate the risks associated with HBC for their overall health and wellbeing (192, 193).

Failing to provide accurate, correct, or inclusive information due to bias or negligence would be a disregard for women's autonomy in making health-related decisions and could hinder their ability to have healthy and fulfilling sexuality (78, 80, 81, 194). Lack of information may also lead to potential or definite therapeutic outcomes associated with the medication, leaving individuals without the possibility of opting out. This becomes particularly critical when these effects persist for the rest of their lives or for several years. A lack of information exchange undermines trust between healthcare professionals/providers and HBC users (195). This mistrust would be aggravated if there were no other drug alternatives, treatments, or fertility control approaches offered to women (196, 197).

Several recommendations have been proposed to prevent information avoidance and facilitate the dissemination of potential adverse effects (31). These include completely specifying them in the labeling, incorporating a black box warning or flags to indicate a potential increased risk in specific pathologies, or considering their removal from the market when there is sufficiently consistent evidence. Additionally, training courses may be provided to healthcare professionals to improve their service (198–203) and knowledge of alternative approaches to reach “coercion-free” contraception (197). Professionals in the scientific, commercial, or healthcare areas cannot withdraw their commitment to providing complete and truthful drug safety and efficacy information, including their adverse effects (21, 204). Online and network sources would not suffice due to the risks of unverified information and the effect that the validity of the content may have on patients' health (205–207). In reality, good quality women's health care demands that health professionals seek communication channels for accurate, reliable, precise, balanced, unbiased, and personalized information to answer the questions raised by women using contraceptives (193, 194, 197) and to prevent HBC users from avoiding gathering information that could threaten their health, wellness, or other interests (208). This attitude would secure women's right to personal autonomy, recognize their dignity, and desire to feel respected, and ensure an effective patient–physician relationship (192, 196).

### 4.2. Scientific language

If controversy regarding HBC adverse effects exists, Brabaharan et al. took a position completely contrary to previously published scientific evidence, stating, “the associations between hormonal contraceptive use and cardiovascular risk, cancer risk, and other major adverse health outcomes were not supported by high-quality evidence” (209). The foundation for such a categorical statement lies in the reinterpretation of the *p*-value (*p*) as a statistical criterion (210–212). The authors define “quality of evidence” based on four arbitrary categories: class 1, a “convincing” value supported by *p* < 0.000001 plus objective and subjective criteria; class 2, with “highly suggestive” evidence with *p* < 10^−6^; class 3, with a “suggestive” quality value and *p* < 0.001; and lastly, class 4, with a poor quality of evidence and *p* < 0.05. Then, the authors require *p* < 10^−6^ to claim statistically significant effects of any intervention rather than the generally accepted *p* < 0.05 value (212–215). This leads to different interpretations (216), including “no-effect” unless the intervention reaches *p* < 10^−6^ value: results without *p* < 10^−6^ would be considered irrelevant, dismissing thousands of studies carried out over decades of research. Taking a *p*-value of < 10^−6^ excludes the vast majority of studies reporting any HBC-related adverse effect. However, the study does corroborate 30 statistically significant (*p* < 0.05) associations with increased risk of adverse effects: cardiovascular (thromboembolism), cancer (breast, cervical), hypertension, Crohn's disease, ulcerative colitis, suicide, and higher triglyceride and cholesterol levels in women with polycystic ovary syndrome, among others. Similarly, using the same *p*-value (0.05), they identified 10 associations suggesting risk reduction, including cancer (glioma, colon, kidney, and ovarian) and other pathologies (209).

Thus, arbitrary changes in the reference system lacking rigor not only show contempt for previous studies but may lead to scientific relativism capable of endorsing any preconceived idea (217) and therefore put women's health at risk due to a lack of objective clinical assessment.

### 4.3. The social and individual impact of contraception

The widespread use of HBC over the last 60 years has brought about a radical change in the perception of sexual relations for women and couples (26). It has contributed to the emergence of a new social culture characterized by sexual freedom, which is often detached from the biological and transcendent nature of human sexual behavior rooted in the anthropological dimension of sexual intercourse (218). This viewpoint has created conflicts with long-established cultural and religious beliefs, as well as with women's own understanding of sexual health (219). The growing social acceptance of contraception has led to increased tensions regarding women's autonomy and the shared decision-making process within couples regarding birth control (or who sets the sexual control within a couple), sexual satisfaction, libido, and interest (197, 218). Moreover, this shift has resulted in significant injustices due to policies focused on “sexual functioning” and emphasizing risk-free pregnancy (218, 219), particularly among vulnerable populations (193).

A global study (93) that examined adolescents (116,820 boys and girls) aged 12–15 years from 38 countries (excluding the EU and North America) has revealed that 8.8% of boys and 9.3% of girls reported attempted suicides. Among those adolescents who are engaged in sexual intercourse, the rate was 19%, and for those with multiple partners, the rate was 28.7% (RR 1.58, 95% CI: 1.27–1.96). Among other factors, the authors identified “impersonal sex,” life dissatisfaction, and a lack of psychological maturity to integrate sexual intercourse (93). These findings might help mitigate the high rate of suicide and suicide attempts observed among young women using HBC (91, 92, 100, 188, 220). Additionally, the US Centers for Disease Control and Prevention (CDC) (221) has shown the negative impact of HBC on relationships, including increased divorce rates with associated consequences for children (poverty, education, and so on). The CDC has also noted the link between hormonal contraception and abortion, which can have negative effects on women (222), as well as the association with abuse and violence (223).

These results highlight the need for studies that go beyond mere descriptions, delve into other dimensions of sexuality beyond the physiological phenomenon, and seek solutions that prevent the deconstruction and fragmentation of natural human procreation through chemical substances. This reconfiguration of an individual's inner ethical structure may lead to a decline in sexual health and satisfaction for both men and women, particularly with prolonged use of HBC (76, 78). Resolving this issue requires understanding the meaning of human sexuality from an anthropological perspective and striving for a satisfactory and fulfilling sexual experience within a couple. Merely focusing on the physical act of genital union may not fully integrate the intimate relational expression of the individuals involved, thereby limiting the potential for complete sexual satisfaction. This could be attributed to the intertwining feelings of trust, safety, tenderness, and the endorsement of women's femininity, among others (224, 225).

### 4.4. Economic impact

Reducing medication-related problems continues to be a significant challenge in healthcare (226) due to the additional burden on patients and the associated costs to the healthcare system, including hospitalizations (227–229). Various efforts have been made to address this issue, such as implementing programs to identify drug–drug interactions, conducting rational analyses of polypharmacy, and optimizing prescription patterns (230, 231).

The economic burden of the widespread use of HBC was assessed by analyzing data from HBC users aged 15–49 years, as provided by the US CDC (221). The analysis specifically focused on excess adverse drug reactions (ADR) resulting from the increased RR associated with HBC use (223). Taking a conservative approach (the lowest RR for each ADR), it was estimated that “over 1.04 million women have developed diseases or disorders linked to the use of hormonal contraceptives, with costs to society of over US$16 billion annually.” The highest contributor is breast cancer (US$10B), followed by depression (US$3.35B), Crohn's disease (US$1.9B), cervical cancer (US$1.0B), and others. In addition, hyperthyroidism and uterine and ovarian cancers were considered to cause a reduction in cases and, consequently, cost savings for the healthcare system (-US$1.45B).

### 4.5. Environmental impact

The widespread use of HBC has led to the release of HBC molecules (natural and synthetic) and their metabolites into the environment through wastewater. This unintended consequence has been described as an “invisible menace” (232) due to its potential environmental impact (223). Environmental surface water drug analysis detected effective concentrations of levonorgestrel, dydrogesterone (233), 17α-ethynylestradiol (234), estradiol (235), progestins, and other steroids (236, 237). The long-term consequences are unknown, although some initial effects include biochemical, histological, and transcriptome changes (235, 237), fertility loss in humans and animals (232, 238), and induced feminization in fish (238). Wastewater treatment has become crucial to eliminating or decreasing HBC surface water concentrations to ensure a no-effect concentration (223).

## 5. Conclusions

The use of HBC involves an artificial state in women that causes physiological and psychological changes, including behavioral consequences (68). Given the utilitarian focus of the HBC information provided (239, 240), key aspects that could identify basic mechanisms underlying the adverse effects have not been researched sufficiently deeply (241, 242). Thus, it is worth asking why requirements for compliance with healthcare quality parameters, including transparency and regulations aimed at protecting women's health, have not been developed or applied. Conversely, we noticed attempts to minimize HBC adverse effects by referring to studies that minimize opposite results (243), seeking explanations outside the scope of the study (244) in populations with a different stratification (97), or disregarding scientifically established criteria (209).

These identified controversies are diverse: first, the denial or minimization of HBC adverse effects; second, their dismissal as non-relevant; third, the modification of the scientific criteria for their analysis; and fourth, information avoidance. All of them portray HBC use as something ordinary, embedded in women's daily routines, ignoring the fact that HBC creates an artificial situation in the woman's physiology. Therefore, it is necessary to explore the potential adverse effects derived from an induced physiological state, taking a holistic approach to women's health. Such an approach may help identify the pharmacological, psychological, and medical interventions to meet the needs that women face during their fertility stage. In addition, the lack of a fitting medical, pharmaceutical, and scientific ethos leads to uncertainty, insecurity, mistrust, and the degradation of the personal–patient relationship in healthcare. Conversely, unbiased, truthful investigations based on the anthropological category of respect may ensure an optimal and respectful approach to women's health.

Finally, when viewed from a historical perspective, it becomes evident that HBC did not meet an unmet medical need but rather became a tool for separating human procreation and fertility from human sexual behavior. This shift places greater emphasis on the demand for the right to sexual health (192, 196, 197). This right encompasses various aspects, including the right to avoid unfair treatment that compromises one's wellbeing and physical and mental integrity, the right to be free from discrimination or hindered access to alternative treatments, and most importantly, the fundamental right to access sufficient and transparent information to make informed and free choices regarding one's fertility and sexual life.

## Author contributions

IS and MM conceptualized the main ideas. IS and MVR executed the main literature search and compilation of the information. IS, MM, and MVR developed the interpretation and relational analysis. IS wrote the manuscript. All authors read, reviewed, and approved the final version of the manuscript.
